# Recent Advances in the Evaluation of the Toxicological and Ecotoxicological Risks of Polymer, Zinc Oxide, Titanium Dioxide and Graphene Oxide Nanoparticles

**DOI:** 10.3390/nano15020097

**Published:** 2025-01-09

**Authors:** Maria Pilar Vinardell

**Affiliations:** Department Biochemistry & Physiology, Faculty of Pharmacy and Food Sciences, Universitat de Barcelona, 08028 Barcelona, Spain; mpvinardellmh@ub.edu

## 1. Introduction

Nanotechnology has substantial potential for advancements in the fields of biology and medicine. Recently, the use of nanomaterials in applications such as sensors, theranostics and drug delivery systems has gained significant attention. Owing to their nanoscale dimensions (1–100 nm) and high surface-to-volume ratio, nanomaterials exhibit unique and highly specific properties in biological environments. However, concerns regarding their safety have emerged, as these materials may induce biological toxicity, including inflammatory responses, cytotoxicity and other adverse effects on human health and the environment [1,2,3].

A critical obstacle to the broader adoption of nanomaterials in medical applications is the absence of standardized toxicity screening protocols to assess the safety of newly synthesized nanomaterials [4,5]; therefore, the use of more advanced techniques that enable high-throughput analysis and minimize interference is necessary [6]. The field of nanotoxicology is currently focused on understanding the influence of the physicochemical properties of nanoparticles (NPs) and developing strategies to protect living organisms. This is achieved through the collection and analysis of data from both in vitro and in vivo studies [7].

Moreover, the health risks posed by micro- and nanoplastics (MNPs) remain largely unknown. For this reason, it is important to conduct studies to assess human exposure and determine whether micro- and nanoplastics can cross the intestinal barrier and thereby enter the bloodstream, potentially causing toxic effects [8].

Regarding the ecotoxicological impact of nanomaterials, the freshwater species Daphnia magna (and invertebrate) and Danio rerio (zebrafish) are among the more commonly studied organisms. Many of the biological responses examined, particularly in these species, are related to oxidative stress [9].

Therefore, it is crucial to study the potential risks of nanomaterials to both human health and the environment, and Special Issues such as the present one contribute to advancing knowledge in this field.

## 2. An Overview of the Published Articles

Choi et al. (Contribution 1) considered the concerns about pollution caused by microplastics and nanoplastics (MNPs) and its potential impact on human health due to internalization by the small intestine. They studied the behavior of MNPs with in vitro human intestine models. They used 50 nm, 100 nm and 500 nm spherical particles of commercialized polystyrene (PS) and exposed them to a monoculture of Caco-2 cells (a human colorectal adenocarcinoma cell line with epithelial morphology), a co-culture of Caco-2 cells with HT29-MTX-E12 cells (a mucus-secreting subclone from colon adenocarcinoma HT29 cells) and a tri-culture of Caco-2 cells with HT29-MTX-E12 cells and M cells induced by Raji B cells (a lymphoblast cell line). The different in vitro models were used to mimic the human intestine. The MNPs were previously characterized. They demonstrated MNPs’ size-dependent translocations depending on cell types and the importance of the protective role of mucus to minimize the internalization and adverse effects of MNPs. Finally, the authors concluded by expressing the need for long-term studies with better physiological culture models to better understand the toxic mechanism of MNPs.

Ortiz-Román et al. (Contribution 2) evaluated the ecotoxicology of TiO_2_ nanoparticles in zebrafish embryos and eleutheroembryos, determining the lethal concentration of 50% of the population (LC50), the hatching rate, embryo development and chemical analysis of the concentration of TiO_2_ accumulated in the tissue. The test method used to study fish embryo acute toxicity was based on OECD guideline 236, exposing the fish embryo to concentrations of TiO_2_ nanoparticles ranging from 75 to 250 mg/L. After 24, 48, 72 and 96 h, appearance, mortality, development and abnormal behavior were visually inspected and recorded using a stereomicroscope. The results indicated that these nanoparticles did not have a great effect on the mortality and hatching of zebrafish embryos when exposed for 48 and 96 h under the conditions studied, but they did have a great effect on their development with physical malformations at all concentrations tested. The authors highlighted the negative effects on ecosystems.

Casiano-Muñiz et al. (Contribution 3) synthesized, characterized and evaluated the toxicity of ZnO NPs in Artemia salina and zebrafish (Danio rerio) to evaluate the potential ecotoxicological effects of ZnO nanoparticles. Physical malformations were observed after 96 h at 50 μg/mL of exposure. The novelty of this work, as pointed out by the authors, is the use of in vivo models to evaluate total protein content, cytochrome P450 and zinc adsorption in adult zebrafish tissues when exposed to ZnO NPs. The study determined critical lethal concentrations and noted physical malformations in the digestive tract of Artemia salina. Furthermore, zinc oxide nanoparticles were detected in fish tissues following exposure to acute toxicity. This studied highlighted the necessity for further research on zinc oxide nanoparticles to improve the understanding of nanomaterials’ mechanisms and potential long-term impacts, translate findings to implications for human health and contribute to the knowledge in this emerging field.

Bueno Macedo et al. (Contribution 4) designed transferrin (Tf)-conjugated PLGA nanoparticles (NPs) containing an organoselenium compound as an alternative to enhance the efficacy of cancer therapy and reduce the cytotoxicity of the cancer treatments. Active targeting is a strategy that is successful in increasing the efficacy of cancer treatment because it can improve the affinity of NPs to cancer cells. In vitro cytotoxicity studies were performed on different sensitive tumor cell lines and on an MDR tumor cell line. The new Tf-conjugated NPs presented significantly higher antiproliferative activity than their nontargeted counterparts in all cancer cell lines.

Cebadero-Dominguez et al. (Contribution 5) studied the impact of reduced graphene oxide on gastrointestinal digestion in vitro using HepG2, Caco-2 cell lines and 3D intestinal models. The oral route could be a potential way in which these nanomaterials enter the body because of the use of graphene nanomaterials in the food industry, such as in food packaging; their use in medicine; their use in agriculture; and their contamination of water and food. These nanomaterials could also indirectly enter the body due to the ingestion of inhaled material. Digested reduced graphene oxide (rGO) did not produce remarkable cytotoxicity in any of the experimental models employed at the tested concentrations (up to 200 µg/mL), nor was an inflammatory response observed. But undigested rGO has shown cytotoxic effects in Caco-2 cells; therefore, these results suggest that the digestion process could prevent the systemic toxic effects of rGO. The authors postulated that the absence of observed effects may be attributed to the enhanced agglomeration of rGO associated with the digestion process, which could make cellular uptake more difficult or alter rGO cellular interaction.

Mitjans et al. (Contribution 6) performed a comparative in vitro study of the toxicity behavior of zinc oxide (ZnO) nanoparticles, which were 50, 10 nm and micro-sized. The study aimed to investigate the influence of particle size on the toxicity of zinc oxide nanoparticles by characterizing the particles in different media, including cell culture media, human plasma and protein solutions such as bovine serum albumin and fibrinogen.

## 3. Conclusions

In conclusion, the six articles featured in this Special Issue investigated various in vitro and in vivo models to assess the potential toxicological and ecotoxicological effects of different nanomaterials. The nanomaterials examined included metal oxide nanoparticles, specifically titanium dioxide (TiO_2_) and zinc oxide (ZnO), which are commonly used as UV filters in addition to other applications, as well as reduced graphene oxide. Transferrin-conjugated nanoparticles were also studied. While these studies provided valuable insights into the toxicology of nanoparticles, further research is required in all cases to deepen our understanding of their potential impact.
